# Comprehensive myocardial tissue characterization with cardiac magnetic resonance in patients with Churg Srauss Syndrome

**DOI:** 10.1186/1532-429X-17-S1-P347

**Published:** 2015-02-03

**Authors:** Patrizia Pedrotti, Alberto Cereda, Jan Schroeder, Giuseppina Quattrocchi, Angela Milazzo, Cristina Giannattasio, Alberto Roghi

**Affiliations:** 1 Cardiology, Niguarda Hospital, Milano, Italy; 2 Bicocca University, Milano, Italy; 3 Allergology, Niguarda Hospital, Milano, Italy

## Background

Churg-Strauss syndrome (CSS) is a rare systemic necrotizing vasculitis characterized by asthma, hypereosinophilia, cardiac failure, renal damage and peripheral neuropathy. CSS typically develops in three clinical phases, beginning with asthma, followed by tissue eosinophilia and finally in a systemic small-vessel vasculitis. During the late phase, coronary vessels and myocardium are interested leading to a variety of clinical manifestations such as myocarditis, heart failure, pericarditis and coronary vasculitis. Heart involvement is found in over 50% of autopsied CSS patients with evidence of an extremely high mortality in those with cardiac disease left untreated. Cardiac MR has emerged as a promising technique not only for the early diagnosis of cardiac involvement in the setting of this insidious and complex condition, but also for a better risk stratification and tailoring of immunosuppressive therapy.

## Methods

We studied 11 CSS pts with CMR. Standard volumes and LGE-CMR scans were carried out on a 1.5-T scanner (Siemens, Germany), with a dedicated cardiac protocol including right and left ventricular volumes, regional and global wall motion assessment, tissue characterization with T1 and T2-weighted sequences, T1 and T2 mapping and conventional late-enhancement (LGE). MOLLI T1 maps were acquired from 3 short axis slices, extracellular volume was derived from pre and post contrast MOLLI T1 mapping calibrated by blood hematocrit.

## Results

Nine out of 11 pts showed the following cardiac involvement: pericarditis (3/11), STEMI (3/11), heart failure (3/11). We observed a case of severe cardiogenic shock requiring extracorporeal membrane oxygenation (ECMO). Intraventricular thrombus was detected in 3 out of 11 pts, 2 developed cardio-embolic stroke. Comparing pts with CSS with normal subjects indexed volumes tended to be higher (EDVI 91.22 ± 10.34 ml vs. 71.45 ± 5.6 ml, p n.s.; ESVI 46.89 ± 10.47 ml vs. 24 ± 1.55 ml n.s.) and LVEF resulted significantly lower (EF 52.78 ± 6.47 % vs. EF 68.73 ± 5.2 %, p 0.04), with no differences in cardiac mass and right ventricular function. LGE was detected in 9 out of 11 pts (82%), 2 with endocardial LGE, 3 intramyocardial LGE, 3 LGE of papillary muscles, 1 LGE with infiltrative pattern. Higher values of pre-contrast T1 (989.2 ± 16.20 ms vs. 953 ± 7.42, p 0.036) were detected in CSS pts compared to the control group. Patients with EF < 60% had higher LV volumes compared to those with EF ≥ 60% (EDVI 116 ± 12 ml vs 70 ± 6 ml, p=0.004; ESVI 77 ± 10 ml vs 22 ± 2 ml, p< 0.001; EF 34 ± 5 vs 69 ± 2, p< 0.001) and higher ECV compared to healthy subjects (ECV 0.30 ± 0.01 vs. 0.26 ± 0.01 %, p=0.04).

## Conclusions

Patients with CSS have lower LVEF and higher pre-contrast T1 values. LGE was positive in the majority of patients, most often with an endocardial layer pattern. In above 25% of patients a left ventricular thrombus was detected by CMRI.

## Funding

None.

